# Development and validation of mRNA expression-based classifiers to predict low-risk thyroid tumors

**DOI:** 10.3389/fendo.2025.1600815

**Published:** 2025-07-16

**Authors:** Allan Golding, David Bimston, Emma Namiranian, Ellen Marqusee, Gabriel Correa, Evana Valenzuela Scheker, Ruochen Jiang, Yangyang Hao, Mohammed Alshalalfa, Jing Huang, Joshua P. Klopper, Richard T. Kloos, Sara Ahmadi

**Affiliations:** ^1^ Memorial Healthcare System, Interventional Endocrinology, Hollywood, FL, United States; ^2^ Memorial Healthcare System, Endocrine Surgery, Hollywood, FL, United States; ^3^ Brigham and Women’s Hospital, Endocrine, Diabetes and Hypertension, Boston, MA, United States; ^4^ Veracyte, Research Discovery, South San Francisco, CA, United States; ^5^ Veracyte, Medical Affairs, South San Francisco, CA, United States

**Keywords:** thyroid nodule, thyroid cancer, Afirma, molecular diagnostics, thyroid tumor prognosis, machine learning

## Abstract

**Background:**

Molecular variants and fusions in thyroid nodules can provide prognostic information at a population level. However, thyroid cancers harboring the same molecular alterations may exhibit diverse clinical behavior. Leveraging exome-enriched gene expression analysis may overcome the limitations seen in models based on a small number of point mutations or fusions. Here, we developed and validated mRNA-based classifiers with high negative predictive values to preoperatively rule out thyroid tumor invasion and lymph node metastases.

**Materials and methods:**

In this retrospective cohort study, histopathology reports from the Afirma Genomic Sequencing Classifier (GSC) algorithm training and consecutive thyroid cancer patients with Bethesda III–VI thyroid nodules in clinical practice (total 697 and ~50%, respectively) were scored for invasion and metastases. mRNA expression-based classifiers were developed utilizing literature-derived signatures as well as differentially expressed genes between samples with or without clinically significant invasion/metastases as the basic building blocks. Machine learning algorithms were employed to develop the final candidate classifiers. The final locked classifiers were validated on a retrospective cohort of 259 patients with Afirma testing who had thyroid surgery and had invasion and metastasis scores assigned based on histopathology while blinded to the classifier results.

**Results:**

A total of 697 (88% female) patient Afirma samples and scored histology reports were used for classifier development. In development, patients had a median age of 51 years. Ten percent of samples were assigned a high risk for invasion label, and 11.3% were assigned a high risk for lymph node metastasis (LNM) label. A low-risk invasion classifier result was assigned to 41.3% of the cohort with a negative predictive value (NPV) of 97.6%, and a low-risk LNM classifier result was assigned to 49.8% of the cohort with an NPV of 98.6%. In the validation cohort, made up of 75% women with a median age of 53 years, 51% of the samples were ruled out for high risk for invasion label with a 99% [95–100] NPV, and 53% were ruled out for high risk for LNM label with 100% [97–100] NPV.

**Discussion:**

Gene expression-based classifiers that confidently, preoperatively rule out thyroid tumor invasion and lymph node metastasis may help personalize the surgical approach for individuals, reducing overtreatment, surgical complications, and postoperative hypothyroidism.

## Introduction

Approximately 20%–25% of thyroid nodule aspirates result in The Bethesda System for Reporting Thyroid Cytopathology (TBSRTC) Bethesda (B)III or IV (ITN) cytology (1). Historically, consensus guidelines recommended surgery for a definitive diagnosis of ITN (2, 3). The utilization of transcriptional signatures and the discovery of driver mutations promoting thyroid cancer development and influencing its behavior provided the molecular foundation for improved diagnostic accuracy in ITN (4–6). Molecular diagnostics has moved beyond simply aiding in diagnosis and can provide information on tumor prognosis in thyroid nodules with BIII–VI cytology (7, 8).

The extent of thyroid tumor invasion and that of lymph node metastasis (LNM) are strong predictors of structural disease recurrence (9). Although clinically relevant lateral cervical lymphadenopathy should be visible on neck ultrasound (US) imaging, central LNM and intrathyroidal vascular invasion can be challenging to detect preoperatively. For example, due to imaging interference by thyroid tissue, the diagnostic sensitivity of US for central lymph node metastasis can be as low as 51% (10). Molecular variants and fusions, often categorized as *BRAF*-like, *RAS*-like, and non-*BRAF*-non-*RAS*-like, can provide prognostic and tumor behavior information over a population (11, 12). However, individuals with similar somatic thyroid molecular driver mutations can have vastly different clinical presentations. It is well-known that cancer is not a single mutation event, and intra-tumoral molecular heterogeneity, tumor microenvironment, and transcriptional regulatory alterations may influence cancer behavior beyond the effect of a known driver mutation (13, 14). In a retrospective study of the pathologic outcomes of thyroid nodules with different molecular risk groups, less than half of those with high-risk mutations had American Thyroid Association (ATA) high-risk disease on surgical histopathology, while approximately a quarter were ATA low-risk tumors. Over half of the intermediate-risk mutations had ATA low-risk tumors (15). Therefore, when clinicians plan an intervention to manage thyroid nodules suspected or diagnosed as malignant, these classically described canonical molecular alterations may not provide sufficient patient-specific prognostic information. Novel diagnostic tools may provide missing preoperative information to optimize initial thyroid tumor management.

To help address the clinical challenge of ITN, the Afirma Gene Expression Classifier (GEC) was developed and eventually replaced by the Afirma Genomic Sequencing Classifier (GSC) after clinical and analytical validation (4, 6). The Afirma GSC uses exome-enriched RNA sequencing (RNA-seq) combined with machine learning algorithms to classify nodules and detect molecular alterations that provide clinically meaningful diagnostic and prognostic information from thyroid nodule aspirates (16, 17). Here, we develop novel molecular classifiers to preoperatively predict thyroid tumor invasion (INV) and regional LNM among Bethesda III/IV nodules that are Afirma GSC suspicious and Bethesda V/VI nodules by leveraging the abundant data generated by the Afirma platform.

## Materials and methods

### Training cohorts

In this retrospective cohort study, the initial training cohort was derived from the Afirma GSC algorithm training subjects, composed of thyroid nodule patients recruited for the Afirma GEC and subsequent GSC training studies (consecutively collected from 2013 to 2016). These thyroid nodules were mostly ITN, mostly histologically benign, and generally very low-risk thyroid cancer when malignant (4, 6). Given a need to train on samples with outcomes of interest (tumor invasion and locoregional lymph node metastases), a subsequent cohort from an integrative interventional endocrinology and endocrine surgery community practice (Memorial Health, Hollywood, FL, USA) with BIII–VI nodule cytology and malignant final thyroid histopathology was incorporated [consecutive fine-needle aspiration (FNA) dates January 2019 to July 2021]. Together, these cohorts (n = 697) constituted the “training cohort” (**Table 1**).

**Table 1:** Clinicogenomic characteristics of the training, validation, and evaluation cohorts.

	Training cohort	Validation cohort	Evaluation cohort
Total (n)	697	259	17,436
Age (median [IQR])	51 [38–60]	53 [39–62]	54 [40–66]
Sex			
Male	152 (21.8%)	65 (25.1%)	4,244 (24.3%)
Female	545 (78.2%)	194 (74.9%)	13,172 (75.5%)
Cytology Bethesda			
III-GSC suspicious	253 (36.3%)	172 (66.4%)	11,767 (67.5%)
IV-GSC suspicious	132 (18.9%)	65 (25.1%)	4,048 (23.2%)
V	112 (16%)	7 (2.7%)	799 (4.6%)
VI	200 (28.7%)	15 (5.8%)	822 (4.7%)
Invasion outcome			
Low risk: no invasion	542 (77.7%)	220 (85%)	
Low risk: minimal vascular invasion (<4 vessels)	85 (12.2%)	31 (12%)	
High risk: extensive vascular invasion (≥4 vessels)	47 (6.7%)	6 (2.3%)	
High risk: extrathyroidal invasion	23 (3.3%)	2 (0.7%)	
Lymph node metastasis			
Low risk: no nodes	558 (80%)	248 (95.7%)	
Low risk: central neck nodes <2-mm tumor deposit and <40% LN involved	60 (8.6%)	4 (1.5%)	
High risk: central neck nodes ≥2-mm tumor deposit or ≥40% LN involved	53 (7.6%)	5 (1.9%)	
High risk: lateral neck nodes	26 (3.7%)	2 (0.7%)	
Histopathology (median tumor size in cm)			
FA	50 (7.2%)	61 (23.5%) (1.8 cm)	
OA	22 (3.1%)	23 (8.9%) (1.8)	
NIFTP	38 (5.4%)	40 (15.4%) (2.2)	
FTC	19 (2.7%)	10 (3.9%) (1.95)	
OC	18 (2.6%)	15 (5.8%) (2.3)	
IFPTC	144 (20.7%)	26 (10%) (2.1)	
PTC	319 (45.8%)	49 (18.9%) (1.4)	
Other	87 (12.5%)	35 (13.5%) (1.5)	
BRAF variant			
*BRAF*V600E	236 (33.9%)	30 (11.6%)	2,073 (11.9%)

Others included rare medullary thyroid cancer, follicular hyperplasia, colloid nodules, nodular hyperplasia, sclerotic nodules, and thyroid lesions of uncertain malignant potential.

FA, follicular adenoma; OA, oncocytic adenoma; NIFTP, non-invasive follicular thyroid neoplasm with papillary-like nuclear features; FTC, follicular thyroid carcinoma; OC, oncocytic carcinoma; IFPTC, infiltrative follicular subtype of papillary thyroid carcinoma; PTC, papillary thyroid carcinoma.

### Validation and evaluation cohorts

After the tumor INV and LNM classifiers were locked, independent cohorts from Memorial Health (n = 63, FNA dates August 2021 to October 2022) and Brigham and Women’s Hospital (n = 196, FNA dates July 2017 to June 2023), all sent consecutively for Afirma testing as part of their routine clinical practice for nodules with BIII–VI cytology, were analyzed as the validation cohort (**Table 1**). These were consecutive samples with local cytology and histopathology interpretations, and treatment decisions based on the local clinician’s discretion with only commercially available Afirma GSC data.

An evaluation cohort of 17,436 consecutive Afirma-resulted ITN GSC suspicious or Bethesda V/VI samples was derived from the Veracyte CLIA laboratory from routine thyroid nodule molecular testing (2017–2020) (18). The INV and LNM classifiers were applied to assess the proportion of samples ruled out for high risk for invasion label and lymph node metastases by Bethesda cytology category, sex, and mutation type (*BRAF*V600E, *RAS*, or no detected expressed alteration).

### Institutional review board approval

Patients recruited for the Afirma GEC development and validation study provided written informed consent (4). The samples subsequently used for the Afirma GSC algorithm training were approved by institutional-specific review boards, Chesapeake IRB 15.02.0009 (now Advarra IRB, Columbia, MD, USA), and Copernicus Group Independent Review Board VER3-15-067 (now WCG IRB, Princeton, NJ, USA) (6). Patient data (including cytology and histopathology reports) from Memorial Health were collected under WCG IRB protocol # DHF 005-044, and Brigham and Women’s patient data were collected under WCG IRB protocol # DHF 005-077.

### Histopathology scoring

A scoring system was applied to the local pathology thyroid histopathology synoptic report (**Table 1**). For tumor INV, if pathology reported vascular invasion of ≥4 blood vessels (or described extensive vascular invasion) or there was any extrathyroidal extension, the sample was labeled high risk. Otherwise, the sample was labeled low risk for tumor INV. For LNM, if the pathology reported ≥2-mm central lymph node deposits or ≥40% of the central nodes resected as malignant, or if there was lateral lymph node thyroid cancer involvement, the sample was labeled high risk. Otherwise, the sample was labeled low risk. Cases without lymph node dissection (Nx) were assigned the low-risk label, as routine preoperative imaging to assess lymph node disease is recommended (19), and the American Association of Endocrine Surgeons guidelines for the surgical management of thyroid disease do not recommend a routine or prophylactic neck dissection. A central neck dissection is only recommended in selected cases with imaging or clinical (macroscopic) lymph node disease (20). The cut point for cancer features was targeted to where the 2015 ATA guidelines’ risk of structural disease recurrence diagram (Figure 4 in the guidelines) bridged from low- to intermediate-risk cancers (9). Therefore, minor extrathyroidal extension received the high-risk label to clearly delineate ATA low-risk disease. The ATA guidelines utilize an absolute lymph node number involved of >5 to distinguish ATA intermediate-risk cancers from low-risk cancers. Given a concern for labeling tumors with five of five or four of four positive lymph nodes as low risk for metastases, central metastatic lymph node ratio (MLNR) criteria were used for risk assessment, as Nam et al. and Seok et al. reported that central compartment MLNR of >30% and ≥36%, respectively, were significantly associated with recurrence (21, 22). In both studies, MLNR above the thresholds described was statistically significant for thyroid cancer recurrence, whereas overall lymph node yield was not. Samples could be high risk for one category and low risk for another. High-risk and low-risk descriptors were solely for labeling and are not intended to correlate with ATA thyroid cancer pathology risk or risk of recurrence (9).

### RNA sequencing and gene expression

RNA-seq data were used to generate gene expression counts. Raw sequencing data (FASTQ file) were aligned to the human reference genome assembly 37 (Genome Reference Consortium) using the STAR RNA-seq aligner. Normalized expression levels were obtained using variance-stabilizing transformation (VST) from the DESeq2 package accounting for sequencing depth and gene-wise variability (23).

For sample quality control, quality metrics were evaluated against prespecified acceptance metrics for total numbers of sequenced and uniquely mapped reads, the overall proportion of exonic reads among mapped reads, the mean per-base coverage, the uniformity of base coverage, and base duplication and mismatch rates. All quality control metrics were generated using RNA-SeQC (24). Only samples that passed the quality criteria were used for downstream analysis. For further details, please see **Supplementary Methods** in Patel et al. (6)

### Classifier development

Histopathology scoring labels (low risk vs. high risk) were used to train machine learning models to classify samples into low- and high-risk categories for invasion and LNM outcomes using both genomic and cytology variables (**Supplementary Table S1**, **Supplementary Figure S1**).

For the invasion classifier, features related to cancer pathway activity, genomic alterations, gene expression, and cytology variables were tested. Pathway/signature scores of 430 gene signature/pathway gene sets from MSigDB were calculated for each sample as described before (25, 26). These pathway scores were used as features for model training. The combinations of several machine learning (ML) models including random forest (RF), penalized generalized linear model (glm), support vector machine (SVM), and several feature engineering methods were evaluated (**Supplementary Figure S2**). Repeated nested fivefold cross-validation (CV) was used for model training, and parameter optimization was used to reduce overfitting and evaluate model performance. Negative predictive value (NPV), the percentage of patients classified as low risk, and score inter-batch reproducibility were the metrics used for selecting the optimal model. The best-performing model was an RF model that used *BRAF* status, nine cancer pathways/signatures, and cytology group as features (**Supplementary Figure S2**, **Supplementary Table S2**). For the LNM classifier, the combinations of ML models and feature engineering methods were evaluated using the expression of individual genes, genomic alterations, and cytology groups as features (**Supplementary Figures S1**, **S3**). A similar repeated nested fivefold cross-validation approach was used to find the best model. The best-performing model was a penalized glm that uses *BRAF* status, cytology group, and the expression of 32 differentially expressed genes (**Supplementary Figure S3**, **Supplementary Table S2**). For classifiers’ reproducibility, 18 samples were used, with each sample run in three different runs with three replicates. These nine replicates/samples were used to calculate inter-batch standard deviation (SD). The inter-batch analytical assessment showed that both classifiers’ scores were reproducible with SD < 5% of the 98% score range [1st percentile–99th percentile]. The final models were retrained on the full training cohort, locked, and then tested in the validation and evaluation cohorts while blinded to the histopathology results.

The classifiers’ cut points were determined using the per-sample median of repeated fivefold CV scores, which resulted in both a high rule-out percent and a high NPV.

## Results

### Training and validation cohort characteristics

There were 379 pathology reports from the Afirma GSC training cohort and 318 pathology reports from Memorial Health that were scored for a total of 697 paired Afirma GSC samples with histopathology outcomes for classifier development (**Table 1**). There were 152 (21.8%) male and 545 (78.2%) female patients aged 9–86 with a mean age of 51 years [interquartile range (IQR): 38–60]. For tumor invasion, 627 were scored as low risk and 70 as high risk. For LNM, 618 were scored as low risk and 79 as high risk. Among those cases labeled high risk for LNM, where only central nodes were positive, the mean number of nodes resected was eight (median 4, range 1–33 [IQR1–3: 2–14]) with a mean MLNR of 0.68. Fifty-five percent of the training cohort was BIII/IV, and 45% was BV/VI. Thirty-three percent of the samples were *BRAF*V600E classifier positive. Among all training samples, the prevalence of high-risk scores for invasion and LNM on the surgical pathology report was 10.0% and 11.3%, respectively (**Table 1**).

The validation cohort included 259 patients, 65 (25.1%) male and 194 (74.9%) female patients aged 16–81 with a mean age of 53 years [IQR: 39–62]. Nodules with BIII/IV cytology and classified as GSC suspicious accounted for 91.5% of the samples, and the rest had BV/VI cytology. Thirty (11.6%) were *BRAF*V600E classifier positive. Eight (3.0%) were scored as high risk for invasion and 7 (2.7%) as high risk for LNM according to the surgical pathology reports (**Table 1**).

### Invasion classifier performance

**Figure 1:**
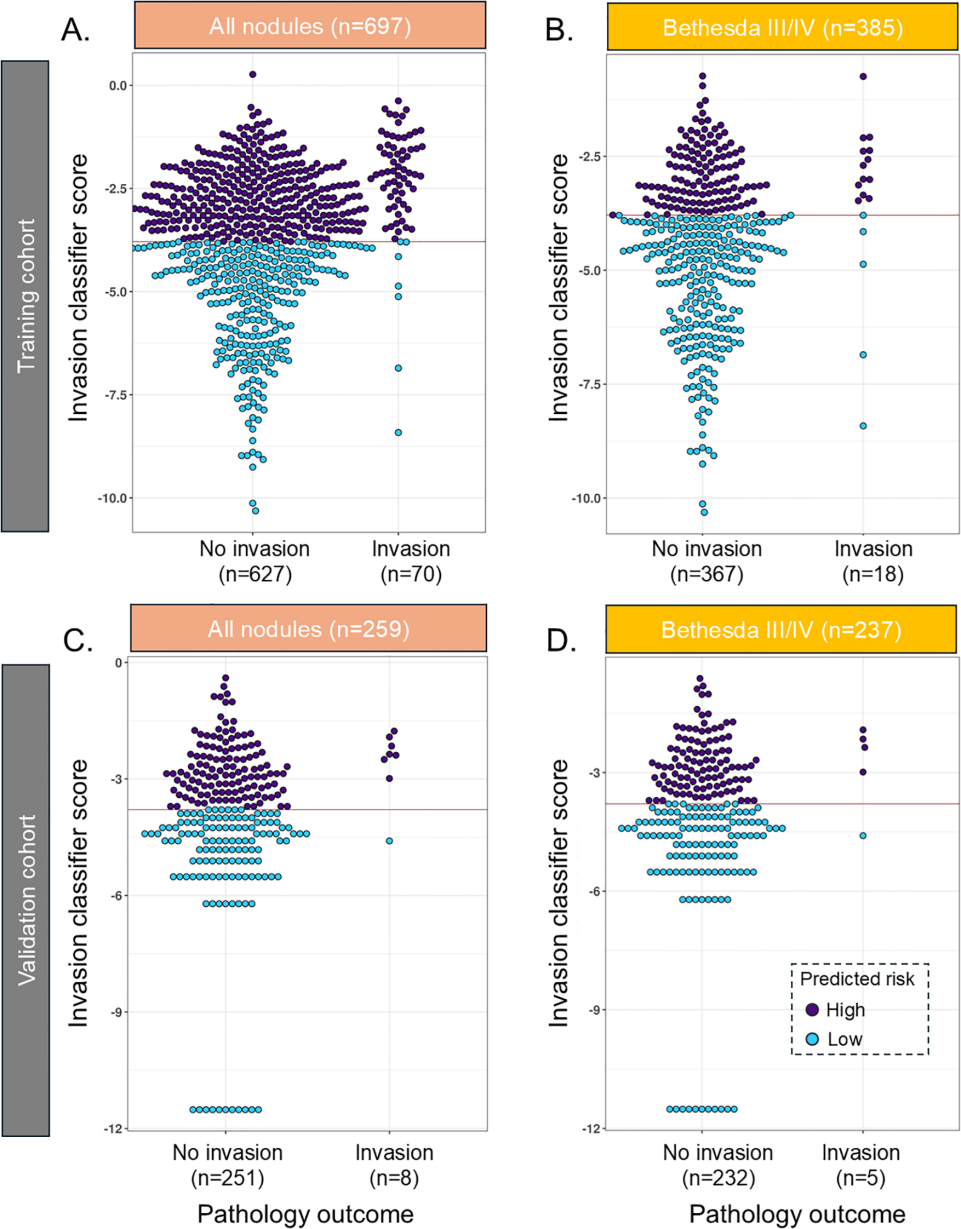
Beeswarm plot of the INV classifier in the training cohort in **(A)** all samples and **(B)** Bethesda III/IV samples and in the validation cohort in **(C)** all samples and **(D)** Bethesda III/IV samples. The red line reflects the cut point where samples below the line are predicted to have a low risk of invasion. INV, invasion.

In the training cohort, the INV classifier had, in fivefold cross-validation, a sensitivity (SN) of 90% [80.5–95.9] and a specificity (SP) of 44.8% [40.8–48.8] and was able to rule out 41.3% of the population for high-risk invasion with a 97.6% NPV (**Figure 1A**, **Table 2a**). In BIII/IV samples (n = 385), 246 (64%) were ruled out for clinically significant invasion with 98% NPV (**Figure 1B**, **Table 2b**). The rule-out percentage was similar in male (44.7%) and female patients (40.4%) (Fisher’s exact test p = 0.35) (**Table 3a**). In samples with BV/VI cytology (n = 312), 42 (13.7%) samples were ruled out (**Table 3a**).

**Table 2:** Classifier performance in the training and validation cohorts.

a.	Classifier performance across all samples		
Classifier	Performance	Training	Validation
INV	Sensitivity	90 [80.5–95.9]	87.5 [47.3–99.7]
	Specificity	44.8 [40.9–48.8]	50.6 [44.2–56.9]
	PPV	15.4 [14.1–16.8]	5.4 [4–7]
	NPV	97.6 [95.2–98.8]	99.2 [95.3–99.9]
	Rule out %	41.30%	49.40%
LNM	Sensitivity	93.7 [85.8–97.9]	100 [59–100]
	Specificity	55 [51–59]	54 [47.6–60.2]
	PPV	21 [19.3–22.8]	5.7 [5–6.5]
	NPV	98.6 [96.7–99.4]	100 [97.3–100]
	Rule out %	49.80%	52.50%

b.	Classifier performance in Bethesda III/IV nodules		
Classifier	Performance	Training	Validation
INV	Sensitivity	72.2 [46.5–90.3]	80 [29–99]
	Specificity	65.7 [60.5–70.5]	54.3 [47.7–60.8]
	PPV	9.3 [7–12.4]	3.6 [2.3–5.6]
	NPV	98 [95.8–99]	99.2 [95.6–99.9]
	Rule out %	63.90%	53.60%
LNM	Sensitivity	73.7 [48.8–90.9]	100 [16–100]
	Specificity	79 [74.4–83]	57.5 [50.8–63.9]
	PPV	15.4 [11.5–20.2]	1.9 [1.7–2.3]
	NPV	98.3 [96.5–99.2]	100 [97.3–100]
	Rule out %	76.40%	56.90%

c.	Classifier performance in Bethesda III nodules		
Classifier	Performance	Training	Validation
INV	Sensitivity	50 [11.8–88]	66.7 [9.4–99]
	Specificity	66.8 [60.5–72.6]	55.8 [47.8–63.4]
	PPV	3.5 [1.6–7.6]	2.7 [1.2–5.9]
	NPV	98.2 [96.1–99.2]	98.9 [94.8–99.8]
	Rule out %	66.40%	57.00%
LNM	Sensitivity	66.7 [34.9–90]	100 [15.8–100]
	Specificity	78.8 [73–83.8]	55.9 [48–63]
	PPV	13.5 [8.9–20]	2.6 [2.2–3.1]
	NPV	97.9 [95.5–99]	100 [96.2–100]
	Rule out %	76.70%	55.20%

d.	Classifier performance in Bethesda IV nodules		
Classifier	Performance	Training	Validation
INV	Sensitivity	83.3 [51.6–97.9]	100 [16–100]
	Specificity	63.3 [54–71.9]	46 [33.4–59]
	PPV	18.5 [13.8–24.3]	5.5 [4.4–6.9]
	NPV	97.4 [91.4–99.3]	100 [88–100]
	Rule out %	59%	44.60%
LNM	Sensitivity	85.7 [42.1–99.6]	
	Specificity	79.2 [71–86]	61 [48–73]
	PPV	18.7 [12.7–26.7]	
	NPV	99 [94–99.8]	100 [91–100]
	Rule out %	75.70%	61.50%

INV, invasion; PPV, positive predictive value; NPV, negative predictive value; LNM, lymph node metastasis.

**Table 3:** Percentage (%) rule out of patients based on INV and LNM classifiers across different subgroups in the training, validation, and evaluation cohorts.

a.	Training cohort (n = 697)	
	INV classifier rule out n (%)	LNM classifier rule out n (%)
Overall (n = 697)	288 (41.3%)	345 (49.8%)
Bethesda		
III (n = 253)	168 (66.4%)	194 (76.7%)
IV (n = 132)	78 (59.1%)	100 (75.7%)
V (n = 112)	20 (17.8%)	45 (40%)
VI (n = 200)	22 (11%)	6 (3%)
BRAFV600E+ (n = 236)	26 (11%)	2 (1%)
Sex		
Male (n = 152)	68 (44.7%)	80 (52.6%)
Female (n = 545)	220 (40.4%)	264 (48.5%)
Histopathology		
FA (n = 50)	38 (76%)	47 (94%)
OA (n = 22)	14 (63.6%)	22 (100%)
NIFTP (n = 38)	27 (71.1%)	33 (86.8%)
FTC (n = 19)	10 (52.6%)	18 (94.7%)
OC (n = 18)	8 (44.4%)	16 (88.9%)
IFPTC (n = 144)	86 (59.7%)	95 (66%)
PTC (n = 319)	73 (22.9%)	57 (17.9%)

b.	Validation cohort (n = 259)	
	INV classifier rule out n (%)	LNM classifier rule out n (%)
Overall (n = 259)	128 (49.4%)	136 (52.5%)
Bethesda		
III GSC-S (n = 172)	98 (57%)	95 (55.2%)
IV GSC-S (n = 65)	29 (44.6%)	40 (61.5%)
V (n = 7)	1 (14.3%)	0 (0%)
VI (n = 15)	0 (0%)	1 (6.7%)
Genomic group		
RAS+ (n = 54)	29 (53.7%)	39 (72.2%)
BRAFV600E+ (n = 30)	2 (6.7%)	0 (0%)
Sex		
Male (n = 65)	33 (50.8%)	31 (47.7%)
Female (n = 194)	95 (49%)	105 (54.1%)
Histopathology		
FA (n = 61)	36 (59%)	41 (67.2%)
OA (n = 23)	13 (56.5%)	17 (73.9%)
NIFTP (n = 40)	25 (62.5%)	25 (62.5%)
FTC (n = 10)	5 (50%)	8 (80%)
OC (n = 15)	9 (60%)	9 (60%)
IFPTC (n = 26)	14 (53.8%)	17 (65.4%)
PTC (n = 49)	12 (24.5%)	5 (10.2%)
Surgery		
Lobectomy (n = 175)	101 (57.7%)	108 (61.7%)
NTT (n = 83)	27 (32.5%)	27 (32.5%)

c.	Evaluation cohort (n = 17,436)	
	INV classifier rule out n (%)	LNM classifier rule out n (%)
Overall (n = 17,436)	9,295 (53.3%)	7,695 (44.1%)
Bethesda		
III GSC-S (n = 11,767)	6,972 (59.2%)	5,171 (43.9%)
IV GSC-S (n = 4,048)	1,971 (48.7%)	2,363 (58.4%)
V (n = 799)	227 (28.4%)	135 (16.9%)
VI (n = 822)	125 (15.2%)	26 (3.1%)
Sex		
Male (n = 4,244)	2,264 (53.3%)	1,886 (44.4%)
Female (n = 13,172)	7,027 (53.3%)	5,807 (44.1%)
Genomic group		
BRAFV600E+ (n = 2,073)	398 (19.2%)	2 (0.1%)
RAS+ (n = 4,028)	2,330 (57.8%)	3,058 (75.9%)
XA negative (n = 9,129)	5,544 (60.7%)	3,854 (42.2%)

NTT, near-total thyroidectomy; XA, Xpression Atlas; INV, invasion; LNM, lymph node metastasis; FA, follicular adenoma; OA, oncocytic adenoma; NIFTP, non-invasive follicular thyroid neoplasm with papillary-like nuclear features; FTC, follicular thyroid carcinoma; OC, oncocytic carcinoma; IFPTC, infiltrative follicular subtype of papillary thyroid carcinoma; PTC, papillary thyroid carcinoma.

In the validation cohort, the INV classifier had an SN of 87.5% [47–100] and was able to rule out 49.4% with 99.2% NPV and a specificity of 50.6% [44.2–56.9] (**Figure 1C**, **Table 2a**). In BIII/IV samples (n = 237), 127 (53.6%) were ruled out with 99% NPV (**Figure 1D**, **Table 2b**), and in BV/VI samples (n = 22), one sample (4.5%) was ruled out (**Table 3b**). The one false-negative sample (**Figure 1C**) had a lobectomy with final pathology showing a 1.2-cm infiltrative follicular subtype of papillary thyroid carcinoma (IF-PTC) with extrathyroidal extension into the adjacent strap muscle. Completion thyroidectomy was benign.

There was no significant difference in performance when comparing samples with BIII or BIV cytology (**Tables 2c, d**).

### LNM classifier performance

**Figure 2:**
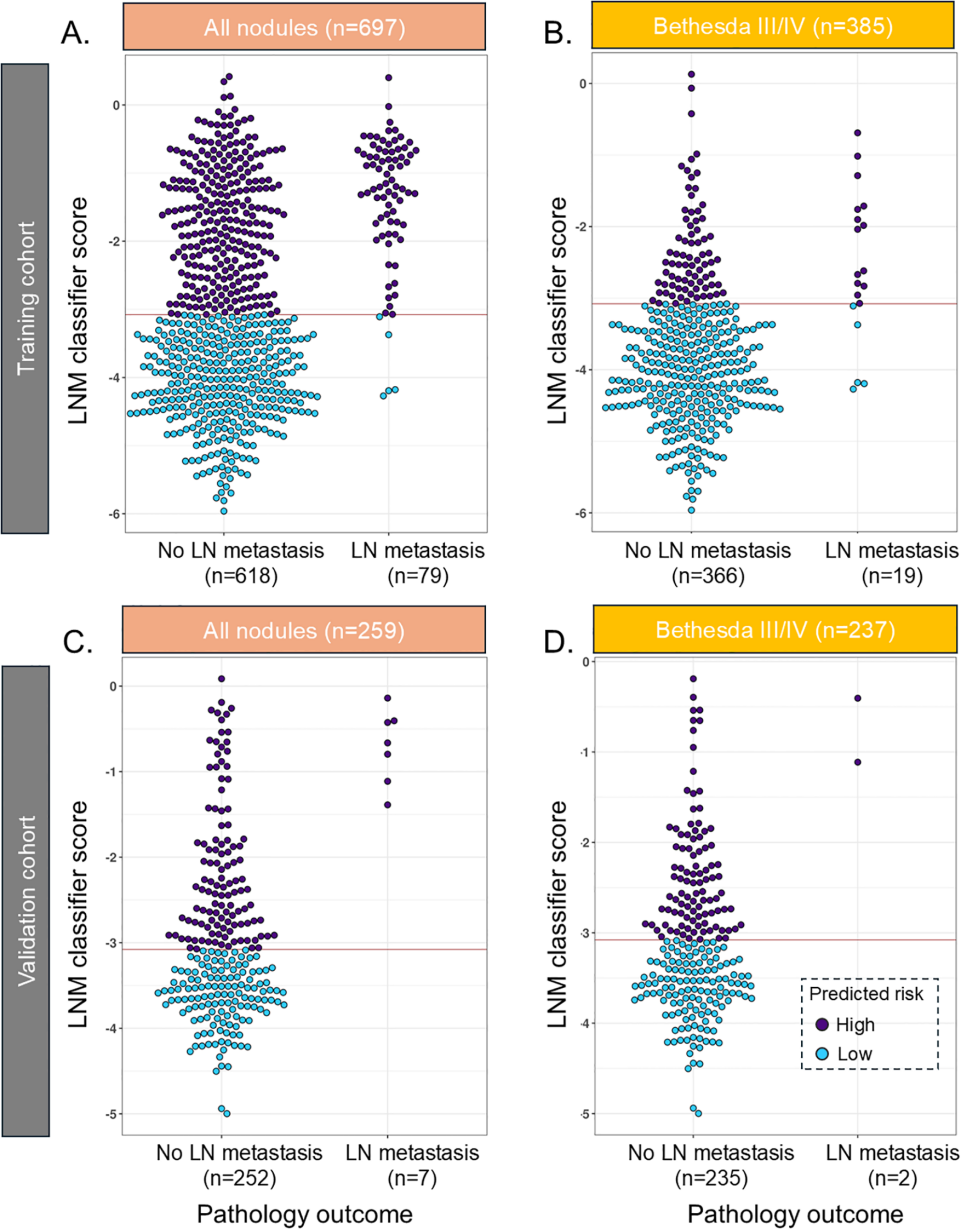
Beeswarm plot of the LNM classifier in the training cohort in **(A)** all samples and **(B)** Bethesda III/IV samples and in the validation cohort in **(C)** all samples and **(D)** Bethesda III/IV samples. The red line reflects the cut point where samples below the line are predicted to have a low risk of lymph node metastasis. LNM, lymph node metastasis.

In the training cohort, the LNM classifier had, in fivefold cross-validation, an SN of 94% [85.8–97.9] and an SP of 55% [51–59] and ruled out 49.8% of the population for high-risk LNM with a 98.6% NPV in the training cohort (**Figure 2A**, **Table 2a**). Of Bethesda III/IV samples (n = 385), 294 (76%) were ruled out for high-risk LNM with 98% NPV (**Figure 2B**, **Table 2b**), and of those with Bethesda V/VI (n = 312), 51 (16.3%) samples were ruled out (**Table 3a**). The rule-out percentage was similar in male (53%) and female patients (49%) (Fisher’s exact test p = 0.41) (**Table 3a**).

In the validation cohort, 44% of the cases had lymph nodes removed, with 14% of those meeting a threshold of at least six nodes removed, which suggested being adequate as a central neck dissection (27). Fifty percent of the local pathology reports did not make any comment regarding lymph nodes, and these were almost exclusively benign cases or non-invasive follicular thyroid neoplasms with papillary-like nuclear features (NIFTP). The LNM classifier had an SN of 100% [59–100] and ruled out 52.5% with 100% NPV and an SP of 54% [44.6–61.6] (**Figure 2C**, **Table 2a**). In BIII/IV samples (n = 237), 135 (57%) were ruled out with 100% NPV (**Figure 2D**, **Table 2b**), and in samples with Bethesda V/VI (n = 22), one sample was ruled out (6.7%) (**Table 3b**).

There was no significant difference in performance when comparing samples with BIII or BIV cytology (**Tables 2c, d**).

### Surgical interventions

The initial surgical intervention was assessed, and all were total thyroidectomy (TT) or lobectomy. In the validation cohort, there were 83 TT (32.5%) (**Table 3b**). Of those with ITN, there were 62 TT (26%). Of all samples with a TT, 16 (19%) had a low-risk invasion classifier alone, 13 (16%) had a low-risk metastasis classifier alone, and 11 (13%) had both low-risk classifiers. Of the 40 (48%) tumors with at least one low-risk classifier, 39 were either histologically benign, NIFTP, or ATA low-risk cancers. The one ATA high-risk cancer was an IF-PTC that had BVI cytology, harboring an *NRAS*:Q61R variant, was >6 cm in size with extensive vascular invasion, and had a correctly assigned INV classifier (not ruled out for INV) and a correctly assigned low-risk LNM classifier (ruled out for LNM) with N0 on final pathology (0/11 nodes).

### Evaluation cohort

An evaluation of 17,346 Afirma GSC-suspicious samples with no clinical outcomes was assessed to compare the tumor classifier scores in an unselected consecutive cohort. These samples were from patients with a median age of 54 years [IQR 40.4–65.9] (**Table 1**). Sex was 75.5% female, and the Bethesda cytology categories were as follows: 67.5% BIII, 23.2% BIV, 4.6% BV, and 4.7% BVI. Overall, 53.3% had a low-risk invasion INV classifier score, and 44.1% had a low-risk LNM classifier score (**Table 3c**). The percentages of samples ruled out by sex, *BRAF*V600E (an Afirma GSC classifier with a limit of detection of >5% variant allele frequency considered as positive) (28), *RAS*, and Xpression Atlas (XA) (17) negative mutation status are shown in **Table 3c**.

## Discussion

Optimal thyroid nodule management requires pre-treatment information regarding the benign or malignant state of a nodule and how it may behave. Clinical, imaging, and cytology features from FNA can provide diagnostic and prognostic information. However, patients rarely have compelling historical or physical exam features suggestive of malignancy, and most thyroid ultrasound assessments result in ATA low- or intermediate-risk classification or American College of Radiology (ACR) Thyroid Imaging Reporting and Data System (TI-RADS) TR3 or TR4, which are not diagnostic (9, 29). Uncertainty may also be present even when high-risk features appear to be present on thyroid ultrasonography. A recent study of oncologic outcomes among patients undergoing surgery after active surveillance for papillary thyroid cancer noted a poor correlation between suspected aggressive US features such as extrathyroidal extension and operative findings where less than one-third of these suspected features on imaging were present on final histopathology (30). ITN cytology leads to uncertainty, and molecular testing can provide both diagnostic and prognostic data, which may guide the extent of surgery if resection is appropriate (20). Molecular testing may also provide valuable prognostic information, informing the appropriate extent of initial thyroid surgery in nodules with BV and BVI cytology (7, 31). However, molecular variants and fusions may not provide sufficient tumor-specific behavioral information. For example, a *BRAF*V600E mutated papillary thyroid cancer can present along a spectrum from an intrathyroidal microcarcinoma to widely metastatic stage IV cancer. Thyroid nodules with RAS mutations may have final histology of benign, NIFTP, or low-risk or high-risk malignancy (32). This presents an opportunity for novel molecular tools that look beyond single-gene mutations to predict tumor-specific behavior and help optimize the initial approach to thyroid nodule and thyroid cancer therapy.

Gene expression profiles utilizing transcriptomic data correlated with pathology outcomes of interest have been used to create prognostic tests for breast and prostate cancers (33–35). Whether other advanced classifier development methods, such as proteomics or the use of single-cell transcriptomics, alone or in combination with bulk sequencing, could be leveraged to develop thyroid cancer prognostic tools will require future studies (36, 37).

The 2015 ATA thyroid nodule and cancer guidelines give clear guidance for when a total thyroidectomy should be performed, including cancers >4 cm, those with gross extrathyroidal extension, and clinical lymphadenopathy (9). For tumors <4 cm without clinically apparent aggressive features, which make up most thyroid cancers, the guidance provided is that a thyroid lobectomy may be adequate, although a total thyroidectomy is reasonable and may be preferred. Despite these guidelines, evidence suggests that patients with cytologically indeterminate nodules and thyroid cancer are likely treated with excessive use of bilateral (total) thyroidectomy (32%–70% of the cases) (38–40). To supplement existing guidelines, preoperative tools that help clinicians accurately de-escalate treatment planning are needed. For low- to intermediate-risk thyroid cancers, studies have shown that survival is the same overall for patients undergoing lobectomy as compared to total thyroidectomy (41). Additionally, thyroid lobectomy results in a lower incidence of early postoperative adverse symptoms including voice changes, tingling, and neuromuscular symptoms (due to parathyroid damage) as compared to total thyroidectomy (42). In terms of longer-term overall quality of life, Yaniv et al. demonstrated that the requirement for levothyroxine after any thyroid procedure was associated with lower quality of life (43). Of course, levothyroxine treatment is required after a total thyroidectomy.

Based on the most recent ATA guidelines, thyroid cancer invasion and regional lymph node metastases are relevant tumor features that predict stage and disease recurrence (9). Thus, these features were incorporated into the classifier training. Given the low prevalence of more aggressive histology in the training cohorts (~10% prevalence of significant invasion or lymph node metastases), classifiers with high NPV were locked. These thyroid tumor classifiers may provide high confidence in performing less aggressive surgery than a total thyroidectomy. Both the INV and LNM classifiers can predict a very low risk (<3%) of clinically significant vascular and extrathyroidal invasion as well as lymph node metastases, and ~50% of the validation cohort was ruled out for these more aggressive pathologic features. The evaluation cohort had a similar rate of low-risk tumor classifier scores, and these were shown to be consistent even when XA was negative for a large panel of genomic variants and fusions, where *BRAF*-like and *RAS*-like molecular risk stratification cannot be invoked (**Table 3c**). Of the patients in the validation cohort who received TT (32.5% overall and 26% of those with ITN), almost 50% had at least one if not both low-risk classifiers, and all but one tumor was either benign or an ATA low-risk cancer. One could hypothesize that a low-risk tumor classifier result may have reduced TT surgeries, although a robust prospective study is required to provide convincing data.

Given the limitation of this retrospective analysis, it is not known if other clinical or patient preference factors dictated the decision to perform a TT. However, those indications, such as contralateral nodules or current levothyroxine treatment, have been described as “soft” indications and are unrelated to expected oncologic outcomes (44). It is possible that if treating physicians and/or patients have highly accurate and reassuring preoperative prognostic indicators, there may be more comfort in performing less aggressive surgery or even monitoring in appropriately selected patients. Importantly, the classifiers described here are not intended to be used in isolation. Additional information includes clinical and imaging features and the Afirma GSC and XA results to provide additional prognostic context.

Here, we demonstrate classifiers that identify less aggressive tumors, regardless of their final histopathology. Ideally, additional classifiers could be developed to predict aggressive thyroid cancer. A barrier to such development is the low prevalence of aggressive thyroid cancer, particularly among those with ITN cytology. For any diagnostic test with a less-than-perfect specificity, a low pre-test prevalence diminishes the positive predictive value that can be achieved (45). Additionally, a test reporting a very high positive predictive value (>95%) for aggressive features may correlate mostly with diseases that already had concerning clinical and ultrasound features. Indeed, in the study of Schumm et al., all patients with high-risk molecular alterations (determined retrospectively) underwent total thyroidectomy and radioiodine ablation based on clinical and ultrasound features, suggesting that the preoperative identification of these genomic alterations may not change management (44).

There are limitations to these classifiers and the current data to support them. The definitions of low-risk and high-risk invasion and lymph node metastasis labels do not necessarily reflect ATA pathology risk, nor do they have formally established long-term clinical outcomes. Additionally, the lack of operative reports describing the approach to lymph node evaluation and the absence of mandatory central neck dissections in training and validation may yield inaccuracies. For example, the low-risk LNM label would be assigned in the absence of any lymph node resection. Still, we believe that thyroid malignancies that are clinically N0 intraoperatively are likely to be at low risk for adverse outcomes. Additionally, in clinical practice, patients with clinical N0 disease receive low American Joint Committee on Cancer stage of disease and ATA risk of structural disease recurrence designation in the absence of aggressive primary tumor features (9, 46). While our validation and evaluation cohort analyses support the locked classifiers, longer-term outcomes will need to be studied. While the NPVs seen were high, the positive predictive values (PPVs) were not high enough to be clinically actionable. Although the accuracy was not different, the proportion of tumors with BV/VI cytology with low-risk classifier scores was low, indicating a need to develop tumor risk classifiers specific to lesions with higher cytologic risk, as a preoperative diagnosis highly suspicious of malignancy may unnecessarily lead to more aggressive surgeries. There are currently no data regarding the risk of recurrence or disease-specific mortality relative to surgical decisions prompted by these classifiers, and these classifiers can only reflect the index lesion undergoing Afirma testing and cannot necessarily account for untested additional foci. Finally, although the training and validation cohorts have some pediatric patients included, a dedicated study evaluating the performance of these classifiers in this population will be necessary given the different molecular profiles of pediatric versus adult thyroid cancer (47). Thus, the classifiers reported here are being made available initially for research use only (RUO) for future investigations when a thyroid tumor either is molecularly suspicious or arises from BV/VI cytology.

In conclusion, the invasion and LNM classifiers developed and retrospectively evaluated in this study indicate high accuracy in predicting low-risk thyroid cancer features. Ultimately, prospective trials assessing how these thyroid tumor INV and LNM classifiers influence surgical interventions and affect clinical outcomes, such as more thyroid lobectomies in lieu of bilateral thyroid resections with no increase in adverse outcomes, will provide necessary insight into their clinical utility.

## Data Availability

The datasets presented in this study can be found in online repositories. The names of the repository/repositories and accession number(s) can be found in the article/**Supplementary Material**.

## Supplementary material

Supplementary Figure 1 Overview of classifiers’ development and validation workflow. Both INV and LNM classifiers were trained using the same machine learning (ML) pipeline with different ML algorithms and features combinations. Repeated five-fold cross validation (CV) was used to evaluate models in training cohort to select the best performing classifier.

Supplementary Figure 2 INV classifiers’ evaluation and best classifier selection. **(A)** Several combinations of machine learning models (generalized linear model (glmnet), hierarchical glm (hier-glmnet), random forest (RF), support vector machine (SVM) with linear or radial kernels) and feature engineering methods (Feature 1-7) were tested to identify the combinations that provide the best performance. Feature engineering methods relied on clustering gene expressions to select representative features from each cluster. To identify the most promising combinations, we evaluated the classifiers using AUC of 5 repeats of 5 fold cross validation (CV). Each box here represents the AUC (from 5-fold CV) values in the 5 repeats. As a control, we added the AUC results of the best single feature to show that models that have more features can show better performance. Results showed that RF and glmnet classifiers gave better performance (AUC) using different feature engineering methods. Overall classifiers that used Feature 7 method (red box) gave slightly higher AUC and more stable scores. **(B)** Since AUC was not the metric, we aimed to optimize to rule out patients with low risk of invasion, we studied the rule out % and NPV on all classifiers that used feature 7 method (red box). Assessing the NPV across different rule-out % showed that the RF classifier preserved the highest NPV across different rule out %. The was the model that was used for further testing in the validation cohort and evaluation cohort.

Supplementary Figure 3 LNM classifiers’ evaluation and best classifier selection. **(A)** We used a similar workflow to the invasion classifier by testing several combinations of feature engineering and machine learning models. We also tested several ensemble models and compared its AUC to individual classifiers. **(B)** Several classifiers were selected for further assessment of the NPV across different rule-out %. We found a single feature that is based on BRAF-RAS score was the most promising. **(C)** To further improve the performance of the BRAF-RAS score, we extracted the genes composing that score and used them as features and then applied different machine learning models. We found that hier-glmnet classifier based on genes can improve the performance compared to BRAF-RAS score a lone. This model was used for further validation and evaluation of the classifier in the validation and evaluation cohorts.

## References

[1] Bongiovanni M Spitale A Faquin WC Mazzucchelli L Baloch ZW. The Bethesda system for reporting thyroid cytopathology: a meta-analysis. Acta cytologica. (2012) 56:333–9. doi: 10.1159/000339959, PMID: 22846422

[2] Baloch ZW Fleisher S LiVolsi VA Gupta PK. Diagnosis of "follicular neoplasm": a gray zone in thyroid fine-needle aspiration cytology. Diagn cytopathology. (2002) 26:41–4. doi: 10.1002/dc.10043, PMID: 11782086

[3] American Thyroid Association Guidelines Taskforce on Thyroid N, Differentiated Thyroid C Cooper DS Doherty GM Haugen BR Kloos RT Lee SL Mandel SJ. Revised American Thyroid Association management guidelines for patients with thyroid nodules and differentiated thyroid cancer. Thyroid. (2009) 19:1167–214. doi: 10.1089/thy.2009.0110, PMID: 19860577

[4] Alexander EK Kennedy GC Baloch ZW Cibas ES Chudova D Diggans J. Preoperative diagnosis of benign thyroid nodules with indeterminate cytology. New Engl J Med. (2012) 367:705–15. doi: 10.1056/NEJMoa1203208, PMID: 22731672

[5] Chudova D Wilde JI Wang ET Wang H Rabbee N Egidio CM. Molecular classification of thyroid nodules using high-dimensionality genomic data. J Clin Endocrinol Metab. (2010) 95:5296–304. doi: 10.1210/jc.2010-1087, PMID: 20826580

[6] Patel KN Angell TE Babiarz J Barth NM Blevins T Duh QY. Performance of a genomic sequencing classifier for the preoperative diagnosis of cytologically indeterminate thyroid nodules. JAMA Surg. (2018) 153:817–24. doi: 10.1001/jamasurg.2018.1153, PMID: 29799911 PMC6583881

[7] Tang AL Kloos RT Aunins B Holm TM Roth MY Yeh MW. Pathologic features associated with molecular subtypes for well-differentiated thyroid cancer. Endocrine Pract. (2020) 27(3):206–11. doi: 10.1016/j.eprac.2020.09.003, PMID: 33655886

[8] Ladenson PW Klopper JP Hao Y Kaushik P Walsh PS Huang J. Combined Afirma Genomic Sequencing Classifier and TERT promoter mutation detection in molecular assessment of Bethesda III-VI thyroid nodules. Cancer cytopathology. (2023) 131:609–13. doi: 10.1002/cncy.22744, PMID: 37544986

[9] Haugen BR Alexander EK Bible KC Doherty GM Mandel SJ Nikiforov YE. 2015 American thyroid association management guidelines for adult patients with thyroid nodules and differentiated thyroid cancer: the American thyroid association guidelines task force on thyroid nodules and differentiated thyroid cancer. Thyroid. (2016) 26:1–133. doi: 10.1089/thy.2015.0020, PMID: 26462967 PMC4739132

[10] Kim E Park JS Son KR Kim JH Jeon SJ Na DG. Preoperative diagnosis of cervical metastatic lymph nodes in papillary thyroid carcinoma: comparison of ultrasound, computed tomography, and combined ultrasound with computed tomography. Thyroid. (2008) 18:411–8. doi: 10.1089/thy.2007.0269, PMID: 18358074

[11] Cancer Genome Atlas Research Network. Integrated genomic characterization of papillary thyroid carcinoma. Cell. (2014) 159:676–90. doi: 10.1016/j.cell.2014.09.050, PMID: 25417114 PMC4243044

[12] Yoo SK Lee S Kim SJ Jee HG Kim BA Cho H. Comprehensive analysis of the transcriptional and mutational landscape of follicular and papillary thyroid cancers. PloS Genet. (2016) 12:e1006239. doi: 10.1371/journal.pgen.1006239, PMID: 27494611 PMC4975456

[13] Garraway LA Lander ES. Lessons from the cancer genome. Cell. (2013) 153:17–37. doi: 10.1016/j.cell.2013.03.002, PMID: 23540688

[14] Rhee JK Lee S Park WY Kim YH Kim TM. Allelic imbalance of somatic mutations in cancer genomes and transcriptomes. Sci Rep. (2017) 7:1653. doi: 10.1038/s41598-017-01966-z, PMID: 28490743 PMC5431982

[15] Liu JB Ramonell KM Carty SE McCoy KL Schaitkin BM Karslioglu-French E. Association of comprehensive thyroid cancer molecular profiling with tumor phenotype and cancer-specific outcomes. Surgery. (2023) 173:252–9. doi: 10.1016/j.surg.2022.05.048, PMID: 36272768 PMC11189592

[16] Walsh PS Hao Y Ding J Qu J Wilde J Jiang R. Maximizing small biopsy patient samples: unified RNA-Seq platform assessment of over 120,000 patient biopsies. J Pers Med. (2022) 13(1):24. doi: 10.3390/jpm13010024, PMID: 36675685 PMC9866839

[17] Angell TE Wirth LJ Cabanillas ME Shindo ML Cibas ES Babiarz JE. Analytical and clinical validation of expressed variants and fusions from the whole transcriptome of thyroid FNA samples. Front Endocrinol. (2019) 10:612. doi: 10.3389/fendo.2019.00612, PMID: 31572297 PMC6749016

[18] Hu MI Waguespack SG Dosiou C Ladenson PW Livhits MJ Wirth LJ. Afirma genomic sequencing classifier & Xpression atlas molecular findings in consecutive Bethesda III-VI thyroid nodules. J Clin Endocrinol Metab. (2021) 106(8):2198–207. doi: 10.1210/clinem/dgab304, PMID: 34009369 PMC8277199

[19] Yeh MW Bauer AJ Bernet VA Ferris RL Loevner LA Mandel SJ. American Thyroid Association statement on preoperative imaging for thyroid cancer surgery. Thyroid. (2015) 25:3–14. doi: 10.1089/thy.2014.0096, PMID: 25188202 PMC5248547

[20] Patel KN Yip L Lubitz CC Grubbs EG Miller BS Shen W. The American association of endocrine surgeons guidelines for the definitive surgical management of thyroid disease in adults. Ann Surg. (2020) 271:e21–93. doi: 10.1097/SLA.0000000000003580, PMID: 32079830

[21] Nam SH Roh JL Gong G Cho KJ Choi SH Nam SY. Nodal factors predictive of recurrence after thyroidectomy and neck dissection for papillary thyroid carcinoma. Thyroid. (2018) 28:88–95. doi: 10.1089/thy.2017.0334, PMID: 29117854

[22] Seok J Ryu CH Park SY Lee CY Lee YK Hwangbo Y. Factors affecting central node metastasis and metastatic lymph node ratio in papillary thyroid cancer. Otolaryngology–head Neck Surg. (2021) 165:519–27. doi: 10.1177/0194599821991465, PMID: 33560176

[23] Love MI Huber W Anders S. Moderated estimation of fold change and dispersion for RNA-seq data with DESeq2. Genome Biol. (2014) 15:550. doi: 10.1186/s13059-014-0550-8, PMID: 25516281 PMC4302049

[24] Wang L Wang S Li W. RSeQC: quality control of RNA-seq experiments. Bioinformatics. (2012) 28:2184–5. doi: 10.1093/bioinformatics/bts356, PMID: 22743226

[25] Liberzon A Birger C Thorvaldsdóttir H Ghandi M Mesirov JP Tamayo P. The Molecular Signatures Database (MSigDB) hallmark gene set collection. Cell Syst. (2015) 1:417–25. doi: 10.1016/j.cels.2015.12.004, PMID: 26771021 PMC4707969

[26] Kishan AU Romero T Alshalalfa M Liu Y Tran PT Nickols NG. Transcriptomic heterogeneity of gleason grade group 5 prostate cancer. Eur Urol. (2020) 78:327–32. doi: 10.1016/j.eururo.2020.05.009, PMID: 32461072 PMC8954568

[27] Lang BH Yih PC Shek TW Wan KY Wong KP Lo CY. Factors affecting the adequacy of lymph node yield in prophylactic unilateral central neck dissection for papillary thyroid carcinoma. J Surg Oncol. (2012) 106:966–71. doi: 10.1002/jso.23201, PMID: 22718439

[28] Hao Y Choi Y Babiarz JE Kloos RT Kennedy GC Huang J. Analytical verification performance of afirma genomic sequencing classifier in the diagnosis of cytologically indeterminate thyroid nodules. Front Endocrinol. (2019) 10:438. doi: 10.3389/fendo.2019.00438, PMID: 31333584 PMC6620518

[29] Tessler FN Middleton WD Grant EG Hoang JK Berland LL Teefey SA. ACR thyroid imaging, reporting and data system (TI-RADS): white paper of the ACR TI-RADS committee. J Am Coll Radiol. (2017) 14:587–95. doi: 10.1016/j.jacr.2017.01.046, PMID: 28372962

[30] Levyn H Scholfield DW Eagan A Boe LA Shaha AR Wong RJ. Outcomes of conversion surgery for patients with low-risk papillary thyroid carcinoma. JAMA otolaryngology– Head Neck Surg. (2024) 150(12):1058–65. doi: 10.1001/jamaoto.2024.1699, PMID: 38749064 PMC11097095

[31] Ali SZ Baloch ZW Cochand-Priollet B Schmitt FC Vielh P VanderLaan PA. The 2023 bethesda system for reporting thyroid cytopathology. Thyroid. (2023) 33:1039–44. doi: 10.1089/thy.2023.0141, PMID: 37427847

[32] Hernandez-Prera JC Valderrabano P Creed JH de la Iglesia JV Slebos RJC Centeno BA. Molecular determinants of thyroid nodules with indeterminate cytology and RAS mutations. Thyroid. (2021) 31:36–49. doi: 10.1089/thy.2019.0650, PMID: 32689909 PMC7864115

[33] Qiu C Wang W Xu S Li Y Zhu J Zhang Y. Construction and validation of a hypoxia-related gene signature to predict the prognosis of breast cancer. BMC Cancer. (2024) 24:402. doi: 10.1186/s12885-024-12182-0, PMID: 38561760 PMC10986118

[34] Spratt DE Zhang J Santiago-Jiménez M Dess RT Davis JW Den RB. Development and validation of a novel integrated clinical-genomic risk group classification for localized prostate cancer. J Clin Oncol. (2018) 36:581–90. doi: 10.1200/JCO.2017.74.2940, PMID: 29185869 PMC6530900

[35] Parker JS Mullins M Cheang MC Leung S Voduc D. Supervised risk predictor of breast cancer based on intrinsic subtypes. J Clin Oncol. (2009) 27:1160–7. doi: 10.1200/JCO.2008.18.1370, PMID: 19204204 PMC2667820

[36] Yang S Liu H Zheng Y Chu H Lu Z Yuan J. The role of PLIN3 in prognosis and tumor-associated macrophage infiltration: A pan-cancer analysis. J Inflammation Res. (2025) 18:3757–77. doi: 10.2147/JIR.S509245, PMID: 40098998 PMC11913039

[37] Guo Q Zhong X Dang Z Zhang B Yang Z. Identification of GBN5 as a molecular biomarker of pan-cancer species by integrated multi-omics analysis. Discov Oncol. (2025) 16:85. doi: 10.1007/s12672-025-01840-9, PMID: 39862327 PMC11762033

[38] Lindeman BM Nehs MA Angell TE Alexander EK Gawande AA Moore FD, Jr. Effect of noninvasive follicular thyroid neoplasm with papillary-like nuclear features (NIFTP) on Malignancy rates in thyroid nodules: how to counsel patients on extent of surgery. Ann Surg Oncol. (2019) 26:93–7. doi: 10.1245/s10434-018-6932-5, PMID: 30341576

[39] Endo M Nabhan F Porter K Roll K Shirley LA Azaryan I. Afirma gene sequencing classifier compared with gene expression classifier in indeterminate thyroid nodules. Thyroid. (2019) 29:1115–24. doi: 10.1089/thy.2018.0733, PMID: 31154940 PMC7141558

[40] Babazadeh NT Sinclair TJ Krishnamurthy V Jin J Heiden KB Shin J. Thyroid nodule molecular profiling: The clinical utility of Afirma Xpression Atlas for nodules with Afirma Genomic Sequencing Classifier-suspicious results. Surgery. (2022) 171:155–9. doi: 10.1016/j.surg.2021.08.058, PMID: 34924179

[41] Adam MA Pura J Gu L Dinan MA Tyler DS. Extent of surgery for papillary thyroid cancer is not associated with survival: an analysis of 61,775 patients. Ann Surg. (2014) 260:601–5. doi: 10.1097/SLA.0000000000000925, PMID: 25203876 PMC4532384

[42] Chen W Li J Peng S Hong S Xu H Lin B. Association of total thyroidectomy or thyroid lobectomy with the quality of life in patients with differentiated thyroid cancer with low to intermediate risk of recurrence. JAMA Surg. (2022) 157:200–9. doi: 10.1001/jamasurg.2021.6442, PMID: 34935859 PMC8696698

[43] Yaniv D Vainer I Amir I Robenshtok E Hirsch D Watt T. Quality of life following lobectomy versus total thyroidectomy is significantly related to hypothyroidism. J Surg Oncol. (2022) 126:640–8. doi: 10.1002/jso.26983, PMID: 35689620 PMC9544480

[44] Schumm MA Shu ML Hughes EG Nikiforov YE Nikiforova MN Wald AI. Prognostic value of preoperative molecular testing and implications for initial surgical management in thyroid nodules harboring suspected (Bethesda V) or known (Bethesda VI) papillary thyroid cancer. JAMA otolaryngology– Head Neck Surg. (2023) 149:735–42. doi: 10.1001/jamaoto.2023.1494, PMID: 37382944 PMC10311424

[45] Leeflang MM Bossuyt PM Irwig L. Diagnostic test accuracy may vary with prevalence: implications for evidence-based diagnosis. J Clin Epidemiol. (2009) 62:5–12. doi: 10.1016/j.jclinepi.2008.04.007, PMID: 18778913

[46] Amin MB Greene FL Edge SB Compton CC Gershenwald JE Brookland RK. The Eighth Edition AJCC Cancer Staging Manual: Continuing to build a bridge from a population-based to a more "personalized" approach to cancer staging. CA: Cancer J Clin. (2017) 67:93–9. doi: 10.3322/caac.21388, PMID: 28094848

[47] Franco AT Ricarte-Filho JC Isaza A Jones Z Jain N Mostoufi-Moab S. Fusion oncogenes are associated with increased metastatic capacity and persistent disease in pediatric thyroid cancers. J Clin Oncol. (2022) 40:1081–90. doi: 10.1200/JCO.21.01861, PMID: 35015563 PMC8966969

