# Supplementary material for: Development and validation of mRNA expression-based classifiers to predict low-risk thyroid tumors
Source: Front Endocrinol (Lausanne). 2025 Jul 16;16:1600815. doi: 10.3389/fendo.2025.1600815 (PMC12307184; doi:10.3389/fendo.2025.1600815)
Supplement: Supplementary file 1 [file Table1.docx]

| Supplementary Table S1: Clinicogenomics characteristics of the training and validation cohorts across tumor behavior outcome groups | | | | | | | | |  |
| --- | --- | --- | --- | --- | --- | --- | --- | --- | --- |
|  | Training cohort (n=697) | | Training cohort (n=697) | | Validation cohort (n=259) | | Validation cohort (n=259) | |  |
|  | INV | | LNM | | INV | | LNM | |  |
|  | Low risk | High risk | Low risk | High risk | Low risk | High risk | Low risk | High risk |  |
| Total (n) | 627 | 70 | 618 | 79 | 251 | 8 | 252 | 7 |  |
| Age (median IQR) yrs | 51 [38-60] | 50 [40-64] | 52 [40-62] | 43 [31-53] | 53 [39-62] | 43 [27-64] | 53 [39-62] | 43 [30-53] |  |
| Sex |  |  |  |  |  |  |  |  |  |
| Male | 126 (20.1%) | 26 (37.1%) | 128 (20.7%) | 24 (30.4%) | 64 (25.5%) | 1 (12.5%) | 64 (25.4%) | 1 (14.3%) |  |
| Female | 499 (79.6%) | 44 (62.9%) | 488 (79%) | 55 (69.6%) | 187 (74.5%) | 7 (87.5%) | 188 (74.6%) | 6 (85.7%) |  |
| Cytology Bethesda |  |  |  |  |  |  |  |  |  |
| III-GSC suspicious | 247 (39.4%) | 6 (8.6%) | 241 (39%) | 12 (15.2%) | 169 (67.3%) | 3 (37.5%) | 170 (67.4%) | 2 (28.6%) |  |
| IV-GSC suspicious | 120 (19.1%) | 12 (17.1%) | 125 (20.2%) | 7 (8.9%) | 63 (25.1%) | 2 (25%) | 65 (25.8%) | 0 (0%) |  |
| V | 89 (14.2%) | 23 (32.9%) | 105 (17%) | 7 (8.9%) | 7 (2.8%) | 0 (0%) | 6 (2.4%) | 1 (14.3%) |  |
| VI | 171 (27.3%) | 29 (41.4%) | 147 (23.8%) | 53 (67.1%) | 12 (4.8%) | 3 (37.5%) | 11 (4.4%) | 4 (57.1%) |  |
| Histopathology |  |  |  |  |  |  |  |  |  |
| FA | 50 (8%) | 0 | 50 (8.1%) | 0 | 61 (24.3%) | 0 | 61 (24.2%) | 0 |  |
| OA | 22 (3.5%) | 0 | 22 (3.6%) | 0 | 23 (9.2%) | 0 | 23 (9.1%) | 0 |  |
| NIFTP | 38 (6.1%) | 0 | 38 (6.1%) | 0 | 40 (15.9%) | 0 | 40 (15.9%) | 0 |  |
| FTC | 17 (2.7%) | 2 (2.9%) | 19 (3.1%) | 0 | 10 (4%) | 0 | 10 (4%) | 0 |  |
| OC | 15 (2.4%) | 3 (4.3%) | 18 (2.9%) | 0 | 15 (6%) | 0 | 15 (5.9%) | 0 |  |
| IFPTC | 136 (21.7%) | 8 (11.4%) | 137 (22.2%) | 7 (8.9%) | 23 (9.2%) | 3 (37.5%) | 26 (10.3%) | 0 |  |
| PTC | 275 (43.9%) | 44 (62.9%) | 255 (41.3%) | 64 (81%) | 44 (17.5%) | 5 (62.5%) | 42 (16.7%) | 7 (100%) |  |
| BRAF variant |  |  |  |  |  |  |  |  |  |
| BRAFV600E | 196 (31.3%) | 40 (57.1%) | 180 (29.1%) | 56 (70.9%) | 27 (10.7%) | 3 (37.5%) | 24 (9.5%) | 6 (85.7%) |  |
| FA: Follicular adenoma, OA: Oncocytic adenoma, NIFTP: Non-invasive follicular thyroid neoplasm with papillary-like nuclear features, FTC: follicular thyroid carcinoma, OC: Oncocytic carcinoma, IFPTC: Infiltrative follicular subtype of papillary thyroid carcinoma, PTC: Papillary thyroid carcinoma | | | | | | | | |  |
|  |  |  |  |  |  |  |  |  |  |
