# Supplementary material for: Development and validation of mRNA expression-based classifiers to predict low-risk thyroid tumors
Source: Front Endocrinol (Lausanne). 2025 Jul 16;16:1600815. doi: 10.3389/fendo.2025.1600815 (PMC12307184; doi:10.3389/fendo.2025.1600815)
Supplement: Supplementary file 2 [file Table2.docx]

Supplementary Table S2: Validation lymph node characteristics

| LNM classifier features | Feature category |
| --- | --- |
| BRAF status | Genomic variant |
| cytology group | Clinical |
| ANKLE2 | Gene expression |
| ANKRD46 | Gene expression |
| ANXA1 | Gene expression |
| ARNTL | Gene expression |
| ASAP2 | Gene expression |
| BID | Gene expression |
| CYP1B1 | Gene expression |
| DCSTAMP | Gene expression |
| DUSP5 | Gene expression |
| EVA1A | Gene expression |
| FCHO1 | Gene expression |
| GABRB2 | Gene expression |
| GBP2 | Gene expression |
| HGD | Gene expression |
| ITGB8 | Gene expression |
| KATNAL2 | Gene expression |
| KCNAB1 | Gene expression |
| LAMB3 | Gene expression |
| LLGL1 | Gene expression |
| LY6E | Gene expression |
| MET | Gene expression |
| PDE5A | Gene expression |
| PDLIM4 | Gene expression |
| PNPLA5 | Gene expression |
| PTPRE | Gene expression |
| RUNX2 | Gene expression |
| SFTPB | Gene expression |
| SFTPC | Gene expression |
| SORBS2 | Gene expression |
| SOX4 | Gene expression |
| SPOCK2 | Gene expression |
| TBC1D2 | Gene expression |

| Invasion classifier features | Feature category | # of genes |
| --- | --- | --- |
| BRAF status | Genomic variant |  |
| cytology group | Clinical |  |
| AMP activated kinase metabolic pathway | Gene signature | 184 |
| Androgen receptor pathway | Gene signature | 9 |
| Cytotoxic lymphocyte signature | Gene signature | 1119 |
| Drug response signature to lovastatin | Gene signature | 2496 |
| Drug response signature to perifosine | Gene signature | 2496 |
| Follicular mesenchymal transition pathway | Gene signature | 7 |
| Immune T cells signature | Gene signature | >10,000 |
| immunophenoscore signature | Gene signature | >10,000 |
| Keratin 5 to 6 | Gene signature | 2 |
