# Supplementary figures and images for: Development and validation of mRNA expression-based classifiers to predict low-risk thyroid tumors
Source: Front Endocrinol (Lausanne). 2025 Jul 16;16:1600815. doi: 10.3389/fendo.2025.1600815 (PMC12307184; doi:10.3389/fendo.2025.1600815)

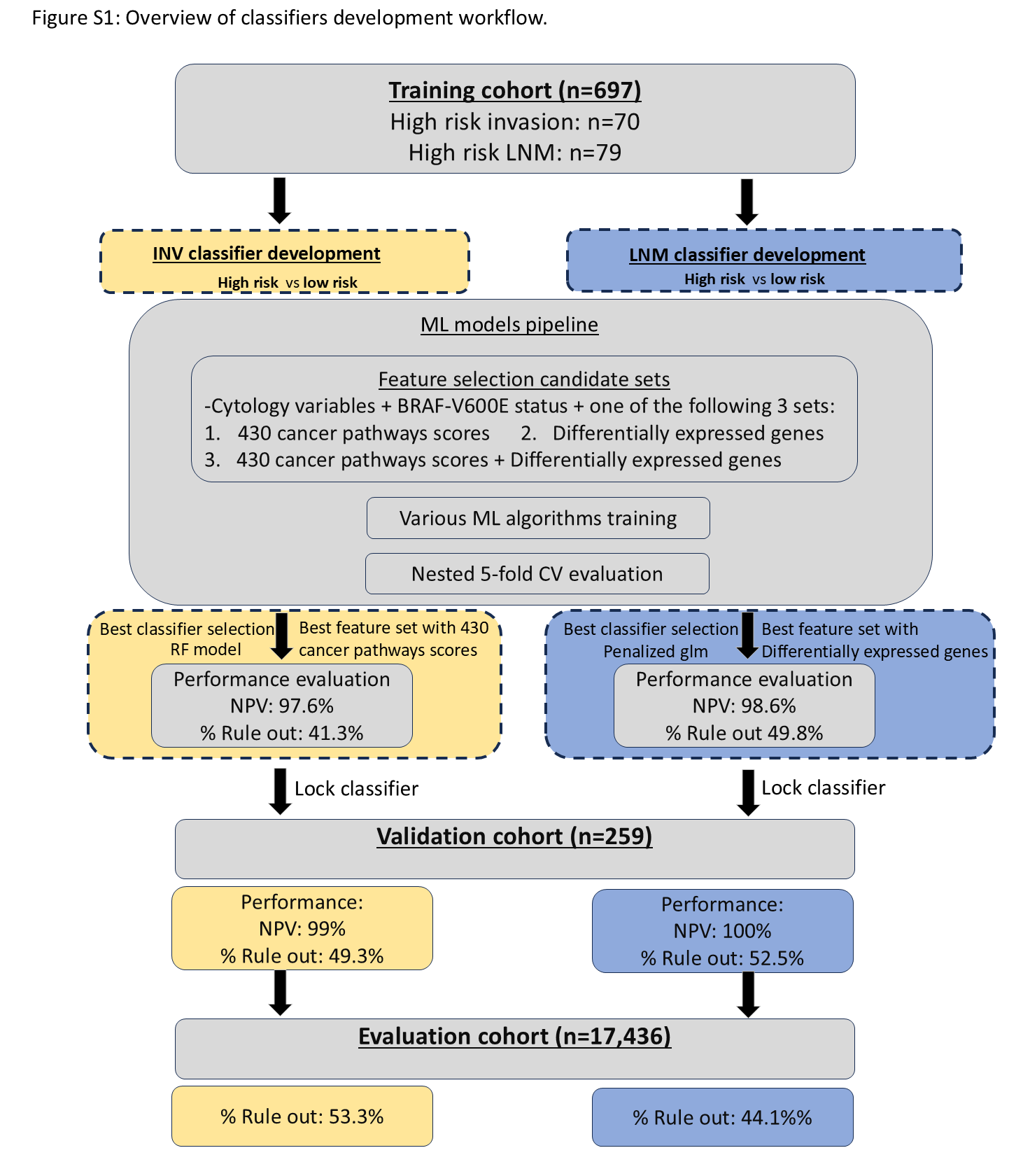

Supplement: Supplementary Figure 1 — Overview of classifiers’ development and validation workflow. Both INV and LNM classifiers were trained using the same machine learning (ML) pipeline with different ML algorithms and features combinations. Repeated five-fold cross validation (CV) was used to evaluate models in training cohort to select the best performing classifier. [file Image1.tif]

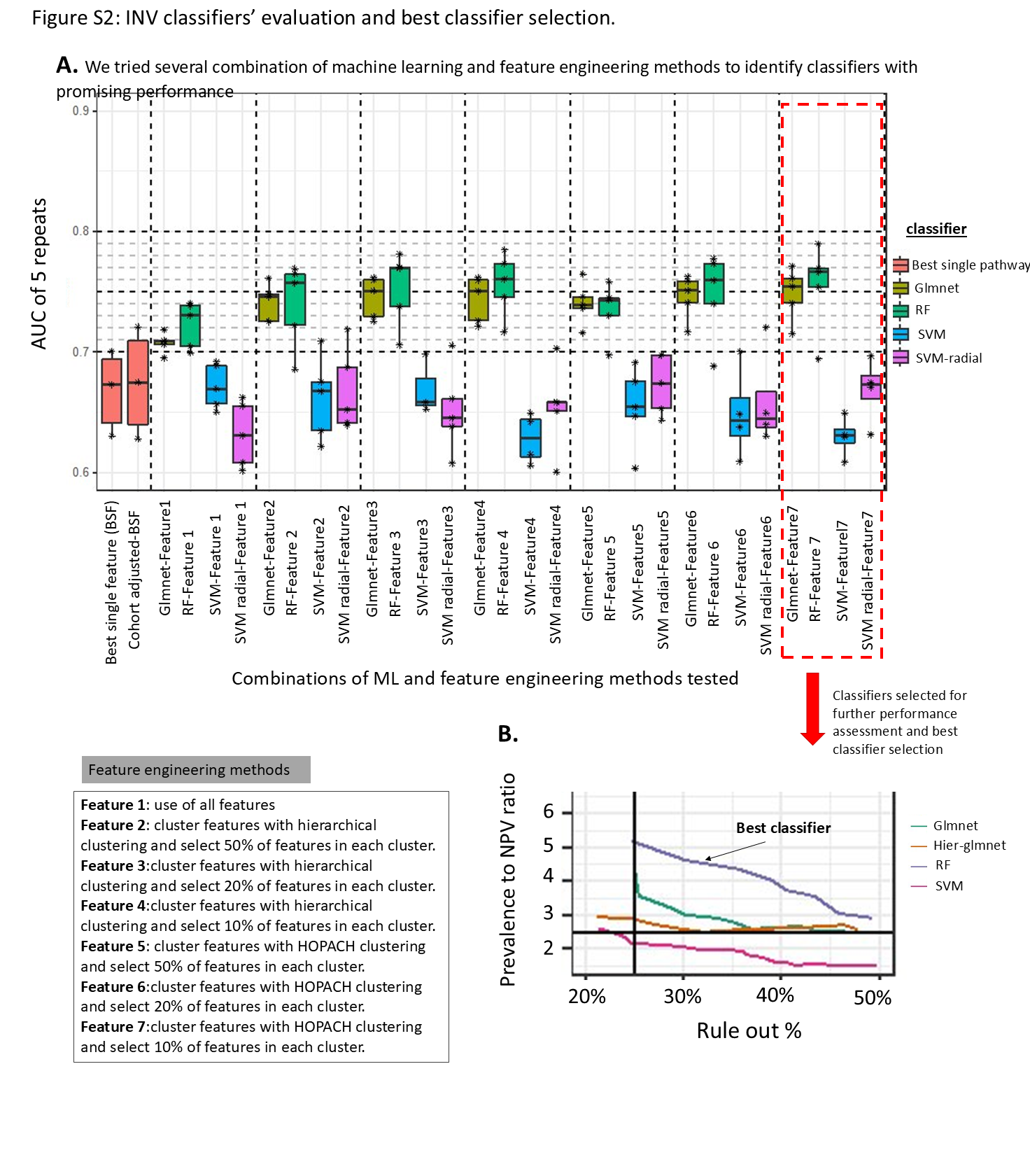

Supplement: Supplementary Figure 2 — INV classifiers’ evaluation and best classifier selection. (A) Several combinations of machine learning models (generalized linear model (glmnet), hierarchical glm (hier-glmnet), random forest (RF), support vector machine (SVM) with linear or radial kernels) and feature engineering methods (Feature 1-7) were tested to identify the combinations that provide the best performance. Feature engineering methods relied on clustering gene expressions to select representative features from each cluster. To identify the most promising combinations, we evaluated the classifiers using AUC of 5 repeats of 5 fold cross validation (CV). Each box here represents the AUC (from 5-fold CV) values in the 5 repeats. As a control, we added the AUC results of the best single feature to show that models that have more features can show better performance. Results showed that RF and glmnet classifiers gave better performance (AUC) using different feature engineering methods. Overall classifiers that used Feature 7 method (red box) gave slightly higher AUC and more stable scores. (B) Since AUC was not the metric, we aimed to optimize to rule out patients with low risk of invasion, we studied the rule out % and NPV on all classifiers that used feature 7 method (red box). Assessing the NPV across different rule-out % showed that the RF classifier preserved the highest NPV across different rule out %. The was the model that was used for further testing in the validation cohort and evaluation cohort. [file Image2.tiff]

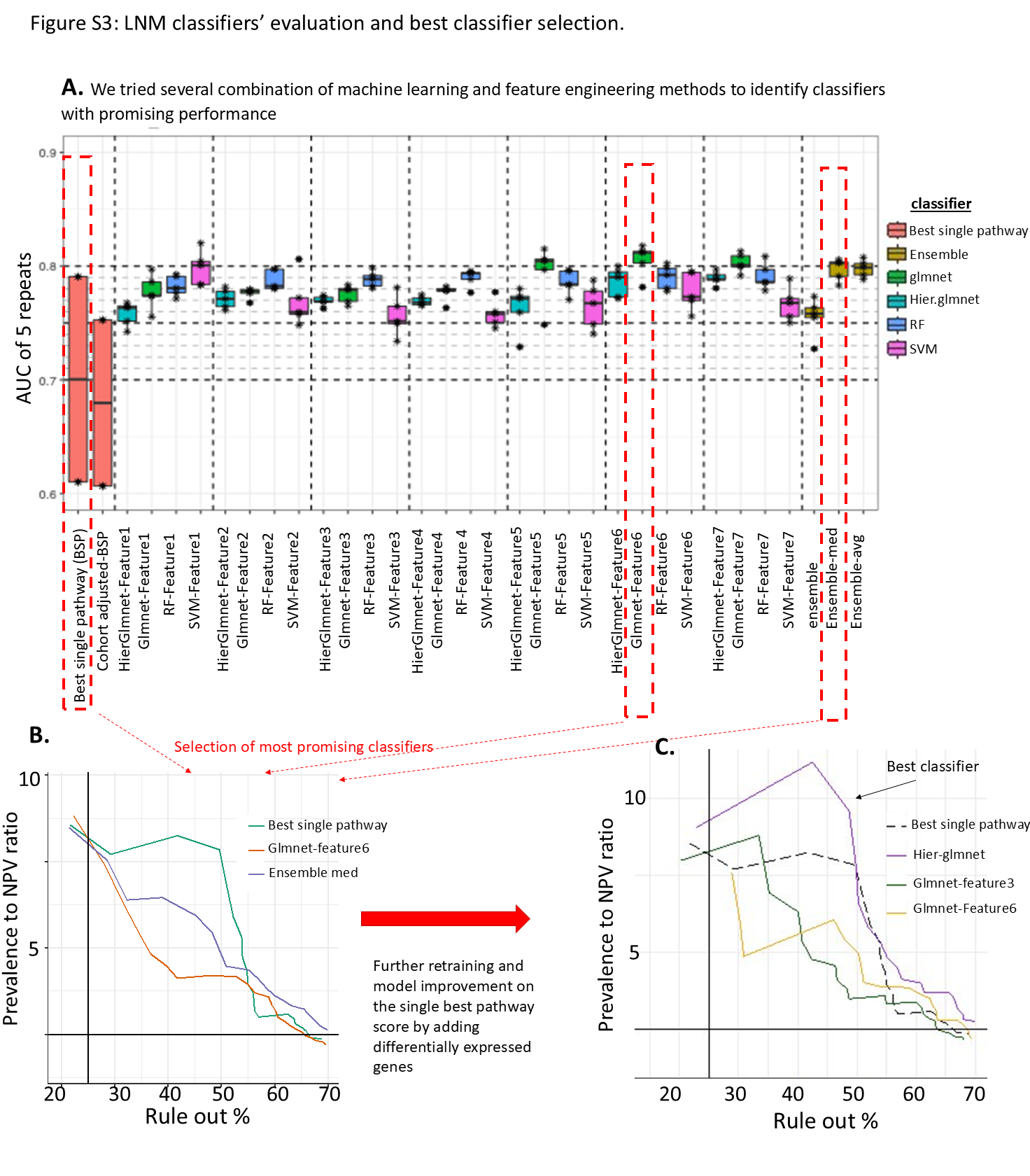

Supplement: Supplementary Figure 3 — LNM classifiers’ evaluation and best classifier selection. (A) We used a similar workflow to the invasion classifier by testing several combinations of feature engineering and machine learning models. We also tested several ensemble models and compared its AUC to individual classifiers. (B) Several classifiers were selected for further assessment of the NPV across different rule-out %. We found a single feature that is based on BRAF-RAS score was the most promising. (C) To further improve the performance of the BRAF-RAS score, we extracted the genes composing that score and used them as features and then applied different machine learning models. We found that hier-glmnet classifier based on genes can improve the performance compared to BRAF-RAS score a lone. This model was used for further validation and evaluation of the classifier in the validation and evaluation cohorts. [file Image3.tiff]
